# Iodine-131 and Thyroid Function

**DOI:** 10.1289/ehp.1307737

**Published:** 2014-02-01

**Authors:** Wenjie Sun

**Affiliations:** Global Health and Environmental Sciences, Tulane University School of Public Health and Tropical Medicine, New Orleans, Louisiana, USA

Ostroumova et al. (2013) reported an association between iodine-131 (^131^I) dose and hypothyroidism in the Belarusian cohort, a cohort of individuals exposed to ^131^I from fallout of the Chernobyl accident when they were ≤ 18 years of age. Ostroumova et al. also examined other thyroid outcomes: hyperthyroidism, autoimmune thyroiditis, serum concentrations of thyroid-stimulating hormone, and autoantibodies to thyroperoxidase.

It may not be appropriate to include participants with other thyroid outcomes in the analysis because those thyroid outcomes could be indirectly associated with exposure. Chernobyl is in an iodine-deficient area (Ishigaki et al. 2001), and the prevalence of goiters among children ≤ 18 years of age has been reported at > 15% in this area (Hatch et al. 2011). Is high prevalence of goiters in the area caused by normal iodine deficiency or by the ^131^I? If the goiters were caused by ^131^I, the relationship between the ^131^I and hypothyroidism is still unclear, even though Ostroumova et al. (2013) stratified the data according to the presence of goiters. Hypothyroidism can also cause goiters (Wilkins et al. 1954); thus, goiter is just a serious hypothyroidism. That could be the explanation for the higher excess odds ratio in the group with goiter compared with the group without goiter shown in Table 3 of Ostroumova et al. (2013). It would have been better for Ostroumova et al. to perform a stratified analysis on the relationship between ^131^I and hypothyroidism based on the normal iodine level of the individual rather than the presence of goiter.

Ostroumova et al. (2013) also claimed that the thyroid radioactivity of individuals from the Belarus cohort was based on a previous study (Stezhko et al. 2004). However, Stezhko et al. (2004) did not provide the details of the individual radioactive iodine measurement. Were the original radioactive iodine measurements generated from a formula or modeled based on food intake or soil contamination, or was the ^131^I exposure level actually measured for each individual? The answer to this question is necessary because the two methods have different credibility. In addition, the exposure described by Stezhko et al. (2004) included ^131^I as well as other radioactive isotopes of iodine, not ^131^I alone. I would like to know whether Ostroumova et al. (2013) separated ^131^I from other radioactive iodine isotopes. Cesium-137 should also be considered as a potential confounder in the relationship between ^131^I and hypothyroidism.
